# 

**Published:** 1888-03-10

**Authors:** 


CONTENTS.

**From Day to Day** - 391

**Words of Consolation.** Chapter LXIX.—The Sufferings of Christ - 392

**The Doctor's Pulpit.**—The Penalties of Weakness - 393

**Medicine Men of the World.** By the Man in the Crowd. Chapter VII. - 394

**Aids to Nursing.** By M. M Basil, M.A. M.B., Author of "The Commoner Accidents to Life and Limb"—Bed and Bedding - 395

**Nursing in East London** - 396

**The National Pension Fund.**—Further Progress - 397

**The British Nurses Association** - 397

**Notes and News** - 398

**Everybody's Page** - 400

**Annotations.**—Cows for Cottagers.—Still on Trial.—Precept and Example at Paddington - 401

**Rachel Challice's Vow.** By Lina Nevill, Author of "A Future on Trust," etc. - 402

**Amusements with Profit for all Ages.**—For Men and Women.—Children's Corner.—Puzzles, etc. - 404

